# Supplementary material for: The association between physical activity intensity and frailty risk among older adults across different age groups and genders: Evidence from four waves of the China Health and Retirement Longitudinal Survey
Source: PLoS One. 2024 Jun 11;19(6):e0305346. doi: 10.1371/journal.pone.0305346 (PMC11166314; doi:10.1371/journal.pone.0305346)
Supplement: S1 Table — (DOCX) [file pone.0305346.s001.docx]

**S1** **Table. Individual deficits of the frailty index.**

| Question | Response | Point |
| --- | --- | --- |
| Comorbidities |  |  |
| 1. Diagnosed with hypertension by a doctor | Yes | 1 |
| 2. Diagnosed with dyslipidemia by a doctor | No | 0 |
| 3. Diagnosed with diabetes by a doctor |  |  |
| 4. Diagnosed with cancer by a doctor |  |  |
| 5. Diagnosed with chronic lung diseases by a doctor |  |  |
| 6. Diagnosed with liver disease by a doctor |  |  |
| 7. Diagnosed with heart attack by a doctor |  |  |
| 8. Diagnosed with stroke by a doctor |  |  |
| 9. Diagnosed with kidney disease by a doctor |  |  |
| 10. Diagnosed with stomach disease by a doctor |  |  |
| 11. Diagnosed with memory-related disease by a doctor |  |  |
| 12. Diagnosed with arthritis by a doctor |  |  |
| 13. Diagnosed with asthma by a doctor |  |  |
| ADL & IADL |  |  |
| 14. Difficulty with running or jogging about 1km | Don't have any difficulty | 0 |
| 15. Difficulty with walking 1km | Have difficulty but can still do it | 0.33 |
| 16. Difficulty with walking 100 meters | Have difficulty and need help | 0.67 |
| 17. Difficulty with getting up from a chair | Cannot do it | 1 |
| 18. Difficulty with climbing several flights of stairs without resting |  |  |
| 19. Difficulty with stooping, kneeling, or crouching |  |  |
| 20. Difficulty with reaching or extending your arms |  |  |
| 21. Difficulty with lifting or carrying weights over 10 Jin(=5kg) |  |  |
| 22. Difficulty with picking up a small coin |  |  |
| 23. Difficulty with dressing |  |  |
| 24. Difficulty with bathing or showering |  |  |
| 25. Difficulty with eating |  |  |
| 26. Difficulty with getting into or out of bed |  |  |
| 27. Difficulty with using the toilet |  |  |
| 28. Difficulty with controlling urination and defecation |  |  |
| 29. Difficulty with household chores |  |  |
| 30. Difficulty with preparing hot meals |  |  |
| 31. Difficulty with shopping for groceries |  |  |
| 32. Difficulty with making phone calls |  |  |
| 33. Difficulty with taking medications |  |  |
| 34. Difficulty with managing money |  |  |
| MMSE & CSI-D |  |  |
| 35. Checking year | Correct | 0 |
| 36. Checking season | Error or cannot answer | 1 |
| 37. Checking date |  |  |
| 38. Checking Day |  |  |
| 39. Checking month |  |  |
| 40. Checking state |  |  |
| 41. Checking County |  |  |
| 42. Checking city |  |  |
| 43. Checking floor |  |  |
| 44. Checking address |  |  |
| 45. Watch correct |  |  |
| 46. Repeat correct |  |  |
| 47. Read correct |  |  |
| 48. Hand correct |  |  |
| 49. Leg correct |  |  |
| 50. Sentence correct |  |  |
| 51. Draw correct |  |  |
| 52. Hammer correct |  |  |
| 53. Store correct |  |  |
| 54. Point correct |  |  |
| CESD |  |  |
| 55. Bothered by things | Rarely or none (<1 day) | 0 |
| 56. Had trouble keeping mind | Some or a little (1-2 days) | 0.33 |
| 57. Felt depressed | Occasionally or a moderate amount of the time (3-4 days) | 0.67 |
| 58. I felt everything I did was an effort (contrary) | Most of the time (5-7 days) | 1 |
| 59. I felt hopeful about the future (contrary) |  |  |
| 60. I felt fearful |  |  |
| 61. My sleep was restless |  |  |
| 62. I was happy (contrary) |  |  |
| 63. I felt lonely |  |  |

ADL: activities of daily living; IADL: instrumental activities of daily living; MMSE: mini-mental state examination; CSI-D: community screening instrument for dementia interviewee part; CESD: center for epidemiologic studies depression scale.
