# Supplementary material for: The association between physical activity intensity and frailty risk among older adults across different age groups and genders: Evidence from four waves of the China Health and Retirement Longitudinal Survey
Source: PLoS One. 2024 Jun 11;19(6):e0305346. doi: 10.1371/journal.pone.0305346 (PMC11166314; doi:10.1371/journal.pone.0305346)
Supplement: S2 Table — (DOCX) [file pone.0305346.s002.docx]

**S2** **Table. Internal consistency of the frailty index.**

| FI | Mean score ± SD | Min | Max | Cronbach Alpha |
| --- | --- | --- | --- | --- |
| Comorbidities (13 items) | 0.242 ± 0.133 | 0.08 | 0.64 | 0.70 |
| ADL & IADL (21 items) | 0.213 ± 0.154 | 0.14 | 0.57 | 0.72 |
| MMSE & CSI-D (20 items) | 0.184 ± 0.173 | 0.05 | 0.53 | 0.73 |
| CESD (9 items) | 0.275 ± 0.104 | 0.22 | 0.62 | 0.67 |
| Total (63 items) | 0.243 ± 0.101 | 0.13 | 0.69 | 0.73 |

ADL: activities of daily living; IADL: instrumental activities of daily living; MMSE: mini-mental state examination; CSI-D: community screening instrument for dementia interviewee part; CESD: center for epidemiologic studies depression scale. A value of Cronbach alpha between 0.7 and 0.9 represented satisfactory internal consistency reliability.
