# Supplementary material for: The association between physical activity intensity and frailty risk among older adults across different age groups and genders: Evidence from four waves of the China Health and Retirement Longitudinal Survey
Source: PLoS One. 2024 Jun 11;19(6):e0305346. doi: 10.1371/journal.pone.0305346 (PMC11166314; doi:10.1371/journal.pone.0305346)
Supplement: S3 Table — (DOCX) [file pone.0305346.s003.docx]

**S3 Table. Internal consistency of the IPAQ (Short Forms) by PA intensity**

| PA intensity | Mean ± SD | Min | Max | Cronbach Alpha |
| --- | --- | --- | --- | --- |
| Light (<600METs/week) | 398 ± 138 | 222 | 427 | 0.79 |
| Moderate (600-3000METs/week) | 1767 ± 463 | 1308 | 2334 | 0.87 |
| Vigorous (>3000METs/week) | 6440 ± 1885 | 3921 | 7483 | 0.91 |
| Total | 2873 ± 628 | 222 | 7483 | 0.85 |

PA: physical activity; IPAQ: international physical activity questionnaire. A value of Cronbach alpha between 0.7 and 0.9 represented satisfactory internal consistency reliability.
