# Supplementary material for: The association between physical activity intensity and frailty risk among older adults across different age groups and genders: Evidence from four waves of the China Health and Retirement Longitudinal Survey
Source: PLoS One. 2024 Jun 11;19(6):e0305346. doi: 10.1371/journal.pone.0305346 (PMC11166314; doi:10.1371/journal.pone.0305346)
Supplement: Attachment — Submitted filename: Response to Reviewers.docx [file pone.0305346.s004.docx]

We appreciate the efforts and comments of the editor and reviewers on our manuscript, “The association between physical activity intensity and frailty risk among older adults across different age groups and genders: evidence from the four waves survey of CHARLS” (PONE-D-24-02757). The comments were helpful in improving the present manuscript. We modified/revised the manuscript, following the reviewer’s comments, concerns, and suggestions. To facilitate the review of these modifications, we have highlighted all changes in RED in the revised manuscript, which we are resubmitting for your consideration.

**Reviewer #1**

**Comment 1****:** Are cognitive function tests being conducted, although answers are mainly based on questionnaires such as the IPAQ?

**Response:** Thank you for your comments. Regarding this question, the FI framework created by Rockwood and Mitnitski was utilized as the dependent variable in our study, including several categories of health problems negatively associated with increasing age. We selected 20 items from the MMSE and CSI-D scales, used to assess cognitive function in older adults, as components of the FI (Table S1). We also conducted a Cronbach internal consistency test (Cronbach Alpha = 0.73) to ensure the robustness of the items (Table S2). The results of the IPAQ test served as the independent variable in this study, representing physical activity intensity. We aim to investigate whether physical activity reduces the risk of frailty in older adults. If so, we aim to determine the optimal intensity of physical activity for older adults of different genders and age groups.

**Comment 2:** Previous studies on the relationship between physical activity and health status at various ages and the relationship between frailty and physical activity have been found in the past. What is the novelty of this study again? Also, please describe and discuss what is different from previous studies.
**Response:** Thank you for your comments. We acknowledge your point of view and recognize that highlighting differences from previous studies can give readers a clearer understanding of the study. Therefore, we have revised and supplemented the original paper in the discussion section (line 235-247).

**Comment 3:** Regarding physical activity, it is believed that it is considerably influenced by the lifestyle of each country. Therefore, we think it would be better to describe the influence of the cultural background of Chinese people.

**Response:** Thank you for your comments. Indeed, as you mentioned, older adults in China usually have a preference for practicing Tai Chi in the mornings at parks and taking part in square dancing activities at community squares in the evenings. This category of physical activities is influenced by traditional Chinese culture, setting it apart from those in other countries. We have already supplemented this aspect in the introduction section (line 65-71).

**Comment 4:** As mentioned in the limitations, the iPAQ questionnaire was used for the physical activity assessment in this study, but would the results be different if an accelerometer or similar instrument were used? I would like to hear your personal opinion.

**Response:** Thank you for your comments. We fully acknowledge the advantage of objective measurement over questionnaire-based assessments in providing accuracy. The use of the IPAQ questionnaire for evaluating physical activity may introduce biases due to its reliance on subjective recollection and estimation. Conversely, accelerometers or similar instruments offer objective, real-time activity data, and there have been studies confirming that the questionnaire might be higher than the objective measurements. However, due to financial constraints, large-scale objective measurements are currently not feasible for us. When utilizing questionnaire data, we first subjected the questionnaire results to consistency testing to ensure a certain level of validity. Moreover, our data stems from a national survey, and with a sufficiently large sample size, we believe the results hold a degree of persuasiveness. In the future, we intend to explore the use of accelerometers or similar objective measurement methods on a small-scale for comparative analysis with questionnaires while ensuring randomization to further enhance the credibility and accuracy of the findings.

**Comment 5:** [Comparing ORs adjusted for covariates shows that vigorous PA within all age 190 groups of 60–64 years (OR, 0.968; 95％ CI, 0.953 to 0.983), 65–69 years (OR, 0.960; 95％ CI, 0.946 191 to 0.973), 70–74 years (OR, 0.964; 95％ CI, 0.947 to 0.981), 75–79 years (OR, 0.957; 95％ CI, 0.939 192 to 0.976), and 80 years and older (OR, 0.938; 95％ CI, 0.918 to 0.957) is the optimal intensity for 193 reducing the incidence of frailty. The moderate PA, after 70 years old, that is, at the age groups 194 of 70–74 years (OR, 0.972; 95％ CI, 0.956 to 0.988), 75–79 years (OR, 0.976; 95％ CI, 0.958 to 0.993), 195 and 80 years and older (OR, 0.960; 95％ CI, 0.946 to 0.973), produces a positive effect on reducing 196 the incidence of frailty.]
→We question whether it is appropriate to use language that suggests a causal relationship of positive effects. Since this is a cross-sectional study, we felt that it was somewhat inappropriate.

**Response:** Thank you for your comments, and we strongly agree with your comments. We recognize that this is an observational study using cross-sectional data and cannot prove the existence of causal relationships or causal directions. We have corrected language that is prone to ambiguity (line 201-204).

**Reviewer #2**

**Comment 1:** The authors state that the coefficient for light-intensity PA is 3.3, the coefficient for moderate-intensity PA is 4.0, and the coefficient for vigorous-intensity PA is 8.0. Usually, low intensity is defined as less than 3 METS, moderate intensity is defined as 3-6 METs, and high intensity is defined as 6 METs or more, but did this affect the results of this study?

**Response:** Thank you for your comments. After revisiting the section of the manuscript regarding the definition of PA intensity coefficients and reviewing additional literature, we have confirmed that the utilization of coefficients 3.3, 4.0, and 8.0 to represent light, moderate, and vigorous intensity, respectively, aligns with the guidelines outlined in the *International Physical Activity Questionnaire Data Processing and Analysis Guidelines*. Additionally, we observed another part of the studies that was consistent with the categorization criteria proposed by the reviewers. Based on the current findings regarding the validity of the IPAQ, it appears that the IPAQ demonstrates better validity in large-scale surveillance studies comparing groups within or between countries rather than at the individual level. When given an adequate sample size, we have reason to believe that the choice of PA intensity subgroup definition would have a negligible impact on the research outcomes.

**Comment 2:** For logistic regression analysis, at least the items that are found to be significant in Table 1 should be included as covariates.

**Response:** Thank you for your comments. Educational background, place of residence, marital status, smoking, and alcohol consumption that were significant in Table 1 were added as covariates in the logistic regression of the adjusted model, whereas no covariates were added in the logistic regression of the crude model. We have corrected the content of the legend (Fig 3 and Fig 4) and elaborated on it.

**Comment 3:** Recent studies have often used objective indicators such as 3-axis accelerometers, but it is necessary to add the validity of classifying physical activity intensity using IPAQ.

**Response:** Thank you for your comments. We deeply appreciate the time and expertise you have invested in reviewing our manuscript, and we recognize the importance of validity when classifying PA intensity using questionnaire measures. Our study using data from the CHARLS national database resulted in our inability to analyze it using the Test-Retest method, the Parallel Form Test method (comparison with objective measuring tools), and others. We ultimately analyzed the internal consistency of the IPAQ questions within the light, moderate, and vigorous intensity groups using the Cronbach coefficients to ensure the validity of the PA intensity subgroups and have added content to this analysis in the Statistical Methods section (line 155-158), the Results section (line 186-189), and the Supporting Information section (Table S3) of the manuscript.

**Comment 4:** Recent studies have reported the usefulness of sedentary time and MVPA. The discussion of this study needs to be updated (ex. PMID: 38349064, PMID: 38332942, PMID: 38219269, PMID: 38030967).

**Response:** Thank you for your comments. We carefully read the latest research developments you provided and reexamined our discussion section. The Mendelian randomization study results have provided new evidence for the causal relationship and direction between PA and reduced frailty risk. Consequently, we have removed the content regarding uncertain causal relationships from the limitation (line 290-294). *~~[Nonetheless, even recognizing that the Biological Gradient (dose-response relationship) is only one component of~~* *~~Hill's Criteria for Causation, we have taken care to avoid asserting any causal relationships and have instead restricted our discussion to the relationship between PA intensity and frailty risk among different age groups and genders.]~~*
